# Obesity: A critical risk factor in the COVID‐19 pandemic

**DOI:** 10.1111/cob.12403

**Published:** 2020-08-28

**Authors:** See Kwok, Safwaan Adam, Jan Hoong Ho, Zohaib Iqbal, Peter Turkington, Salman Razvi, Carel W. Le Roux, Handrean Soran, Akheel A. Syed

**Affiliations:** ^1^ Cardiovascular Trials Unit Manchester University NHS Foundation Trust Manchester UK; ^2^ Faculty of Biology Medicine and Health, University of Manchester Manchester UK; ^3^ Department of Endocrinology Christie NHS Foundation Trust Manchester UK; ^4^ Department of Respiratory Medicine Salford Royal NHS Foundation Trust Salford UK; ^5^ Cardiovascular Research Centre Institute of Genetic Medicine, Newcastle University Newcastle upon Tyne UK; ^6^ Diabetes Complications Research Centre University College Dublin Dublin Ireland; ^7^ Department of Diabetes Endocrinology and Obesity Medicine, Salford Royal NHS Foundation Trust Salford UK

**Keywords:** coronavirus, immune dysfunction, obesity, SARS‐CoV‐2 | bariatric surgery

## Abstract

Obesity is an emerging independent risk factor for susceptibility to and severity of coronavirus disease 2019 (COVID‐19) caused by the severe acute respiratory syndrome coronavirus‐2 (SARS‐CoV‐2). Previous viral pandemics have shown that obesity, particularly severe obesity (BMI > 40 kg/m^2^), is associated with increased risk of hospitalization, critical care admission and fatalities. In this narrative review, we examine emerging evidence of the influence of obesity on COVID‐19, the challenges to clinical management from pulmonary, endocrine and immune dysfunctions in individuals with obesity and identify potential areas for further research. We recommend that people with severe obesity be deemed a vulnerable group for COVID‐19; clinical trials of pharmacotherapeutics, immunotherapies and vaccination should prioritize inclusion of people with obesity.

## 1 INTRODUCTION

Obesity, a known risk factor for respiratory infection, is increasingly being recognized as a predisposing factor in the current coronavirus disease 2019 (COVID‐19) pandemic caused by the severe acute respiratory syndrome coronavirus‐2 (SARS‐CoV‐2).^1^ This has important implications on global health as excess weight, usually represented by a raised body mass index (BMI), affects vast numbers of people worldwide: 39% of adults are overweight (BMI ≥25.0 to 29.9 kg/m^2^) and 13% have clinical obesity (BMI ≥30.0 kg/m^2^) globally.^2^ Western populations have markedly higher rates of obesity: 40% of adults have obesity and another 32% are overweight in the United States,^3^ while in England 29% of adults have obesity and a further 36% are overweight.^4^ The prevalence of obesity in men and women increases with age. In this narrative review, we explore the relationship between excess weight and response to infection with SARS‐CoV‐2 and the severity and complications of COVID‐19, discuss the clinical and public health strategies for managing the risks, and identify research priorities.

## 2 COVID‐19 PANDEMIC

From its first recognition in Wuhan City, Hubei Province in China in December 2019,^5^ SARS‐CoV‐2 has spread globally. The novel coronavirus has similarities in its genetic sequence to SARS‐CoV that caused the severe acute respiratory syndrome (SARS) pandemic in 2003.^6^ They both have the spike (S) protein of coronaviruses. The S protein is primed by cellular serine protease TMPRSS2, facilitating binding with angiotensin‐converting enzyme 2 (ACE2) receptor to gain cellular entry.^6^, ^7^ Compared to SARS‐CoV, the novel SARS‐CoV‐2 has higher affinity for ACE2,^8^ and it is thus more readily transmissible. Despite the relationship between SARS‐CoV‐2 and ACE2, currently there is no evidence to show that ACE‐inhibitors or angiotensin receptor blockers contribute to infection.

The commonest presenting symptoms of COVID‐19 have consistently been fever (98%) and dry cough (70%).^9^ Respiratory manifestations of severe COVID‐19 include pneumonia, pulmonary embolism and acute respiratory distress syndrome (ARDS). All age groups may be infected and median age of hospitalized cohorts varies from 47 to 63 years.^9^, ^10^, ^11^ Infection is more common in men with reported prevalence of 58% to 68%.^10^, ^12^, ^13^ In one intensive care unit (ICU) cohort from Italy, 82% were men.^14^ The higher prevalence of men in hospitalized population needing critical care may also reflect difference in disease severity between the sexes.

The median age of death from the disease was 75 years.^9^ Mortality rates were reported as 2.3% in Hubei Province; however, infection fatality rates were higher elsewhere at, for example, 26% in an Italian ICU.^13^, ^14^ Clinically, COVID‐19 causes lymphocytopaenia (up to 82% of patients), elevations of inflammatory markers including C‐reactive protein (CRP), D‐dimer, interleukins and tumour necrosis factor‐alpha (TNF‐α).^8^, ^9^ It has been suggested that patients more likely to progress to critical disease have higher initial levels of inflammatory markers and D‐dimer.^15^, ^16^ It is recognized that a sub‐group of COVID‐19 patients develop a hyperinflammatory syndrome, or ‘cytokine storm’, with sustained excess production of cytokines and chemokines which may lead to ARDS and multi‐organ failure. These patients require critical care and are less likely to survive.^15^, ^17^ Early indicators of the syndrome may include exceptionally high levels of interleukins, ferritin and D‐dimer from the outset at hospital admission.^18^, ^19^ While obesity is associated with a chronic low‐grade inflammatory state, whether this drives patients with obesity towards a potentially more extreme clinical course is uncertain.

Comorbidities are associated with severe COVID‐19; while they were recorded in 24% to 51% of hospitalized patients,^10^, ^12^, ^15^ they were noted in 68% to 72% of ICU patients.^14^, ^20^ The common comorbidities recorded in reports from China (and elsewhere) included hypertension, cardiovascular disease and diabetes mellitus,^20^ all of which are known to be associated with obesity and indeed obesity itself is increasingly recognized as both a comorbidity and a risk factor.^21^

Many early reports of the current COVID‐19 pandemic do not include anthropometric information; however, more recent reports have identified obesity as a predictor of hospitalization.^16^ In a study from Lille in France, people with obesity were significantly over‐represented among patients admitted to ICU with COVID‐19 compared to non‐SARS‐CoV‐2 respiratory disease in previous years (47.5% vs 25.8%).^22^ Furthermore, the need for invasive mechanical ventilation, a surrogate for the severity of SARS‐CoV‐2, increased with rising levels of obesity, reaching nearly 90% in patients with a BMI > 35 kg/m^2^. Similar associations of obesity and disease severity were observed in hospitalized COVID‐19 patients in China and the United States.^23^, ^24^, ^25^

**FIGURE 1:**
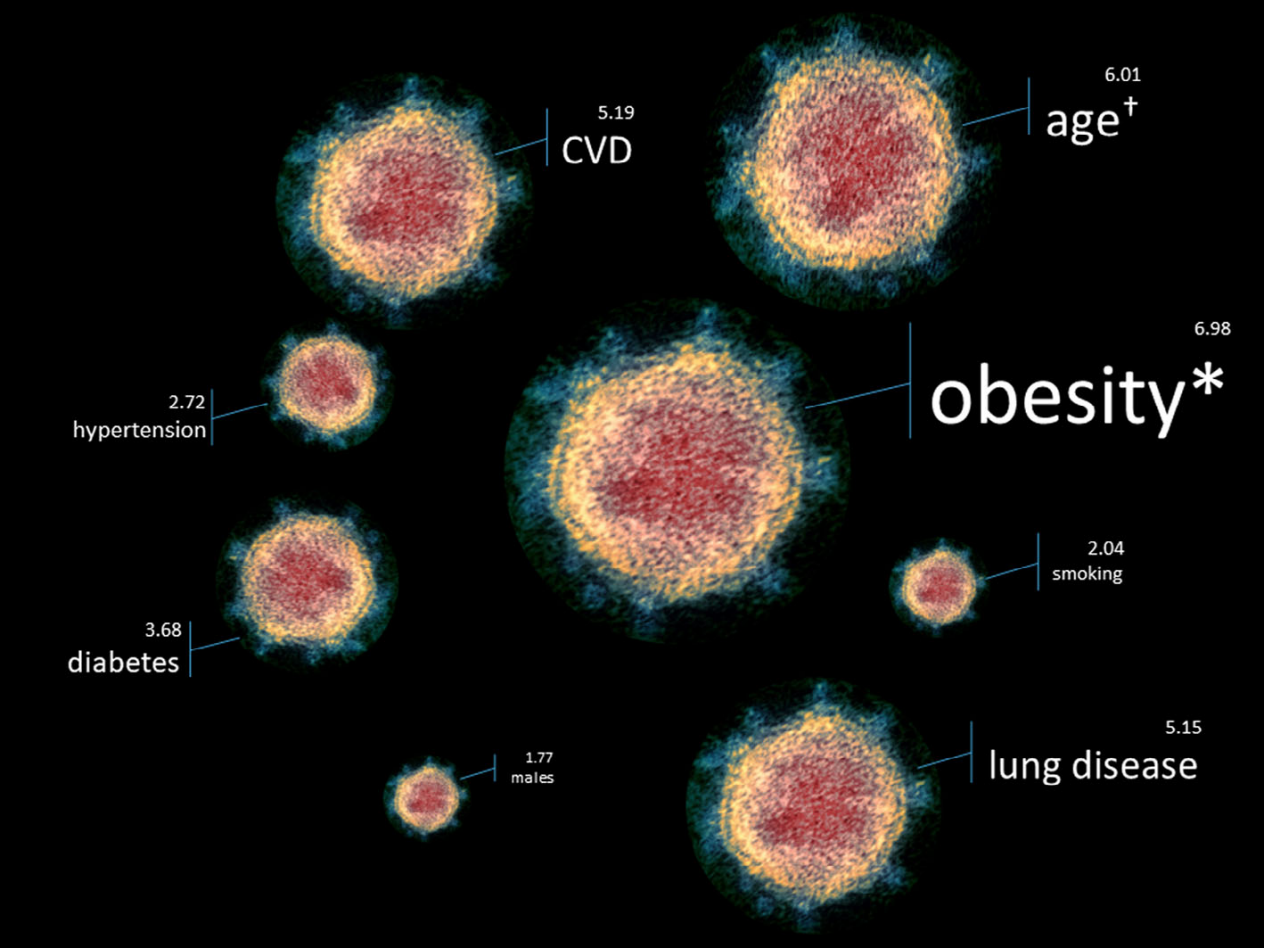
Major determinants of critical illness in COVID‐19. The size of each SARS‐CoV‐2 virion (electron micrograph; credit: NIAID, National Institutes of Health, USA) depicts odds ratio of developing critical vs non‐critical COVID‐19.^26^, ^27^ *Odds ratio for advanced respiratory support in people with body mass index ≥25 kg/m^2^. ^†^Age > 65 years

A systematic review and meta‐analysis of 13 studies with a combined total of 3027 patients with SARS‐CoV‐2 infection found that male sex, age over 65 years, smoking, hypertension, diabetes, cardiovascular disease, and respiratory diseases were associated with worse disease while another meta‐analysis of 14 studies found that obesity was also a predictor of mortality (Figure 1).^26^, ^27^ These findings were similar to those in a UK report of hospitalized COVID‐19 patients.^28^

There is mounting concern of higher incidence of COVID‐19 and worse outcomes in people from black, Asian and minority ethnic communities.^29^, ^30^ While socioeconomic, cultural, or lifestyle factors, genetic predisposition, or pathophysiological differences in susceptibility or response to infection have been mooted,^29^ another factor may be the higher prevalence of metabolic disorders among ethnic minorities of normal weight.^31^, ^32^ Both ‘normal weight obesity’ and ‘metabolically unhealthy normal weight’ have been used to classify normal weight individuals with manifestations of metabolic syndrome such as insulin resistance, dyslipidaemia, and hypertension.^33^, ^34^, ^35^ This cohort of patients are characterized by a metabolically unhealthy fat distribution with increased visceral adiposity but reduced lower‐body fat mass.^36^, ^37^ Furthermore, elevated percentage body fat has been linked with increased cardiometabolic dysregulation and mortality, even among patients of normal weight.^35^

## 3 OBESITY AND RESPIRATORY VIRUSES

Obesity is associated with infection and hospitalization due to respiratory viruses such as coronavirus, influenza, parainfluenza, metapneumovirus and rhinovirus.^38^, ^39^ It was acknowledged to be an independent risk factor in the 2009 H1N1 influenza pandemic.^40^, ^41^ Previous viral pandemics have shown that obesity, particularly severe obesity (BMI > 40 kg/m^2^), is associated with increased risk of hospitalization, ICU admission and fatalities,^42^, ^43^, ^44^ and individuals with obesity have a greater than 6‐fold increase in odds of hospitalization compared to normal‐weight adults.^38^ Emerging evidence indicates that obesity is also a risk factor for the current SARS‐CoV‐2 outbreak.

Obesity contributes to worse disease outcome in viral infections. Obesity has been found to be associated with increased frequency of both upper respiratory tract infections (adjusted OR 1.55) and lower respiratory tract infections (adjusted OR 2.02).^45^ In a mouse model, when diet‐induced obese (DIO) mice and lean mice were infected with mouse‐adapted influenza virus, the DIO mice had greater mortality than the lean mice (42% vs 5.5%).^46^ When two non‐pandemic influenza seasons were examined, an association was found between BMI and disease severity. In the 2003‐2004 and 2004‐2005 influenza seasons, associations of obesity and severe disease were OR 1.14 and OR 1.24 respectively.^41^ Indeed, it was noted during the H1N1 influenza pandemic in 2009 that, whereas there was no evidence that people with obesity had increased susceptibility to infection,^47^ obesity was nevertheless a risk factor for more severe disease and increased pulmonary complications.^41^, ^44^, ^48^ Mortality rates were also significantly higher, especially in those with severe obesity (BMI > 40 kg/m^2^).^39^, ^43^

It is well recognized that in critically ill ICU patients admitted with any cause, patients with obesity have an increased risk of developing ARDS.^49^, ^50^ Unexpectedly, although these patients may have worse morbidity outcomes, their mortality rates are not increased. This ‘obesity paradox’ has been affirmed in meta‐analyses but the mechanisms remain unclear.^51^, ^52^, ^53^, ^54^, ^55^, ^56^ Nonetheless, during the 2009 H1N1 pandemic, this paradox phenomenon was not observed in ICU patients with obesity infected with the virus.^39^, ^43^ It is likely that similar pictures will emerge in the current COVID‐19 pandemic and patients with obesity lose the survival advantage when they contract the virus. Patients with obesity tend to have more comorbidities which may be a contributory factor to poorer outcomes. Furthermore, the mechanical effects of obesity on respiration (discussed further in the next section) may outweigh any potential protection from catabolic effects of severe disease that may accrue from excess energy stores. Another factor may be the dysregulated immune response in patients with obesity to viral infections, particularly impaired T‐cell mediated immunity. This is discussed later in section entitled ‘Obesity and Immune Function’.

## 4 OBESITY AND PULMONARY FUNCTION

**FIGURE 2:**
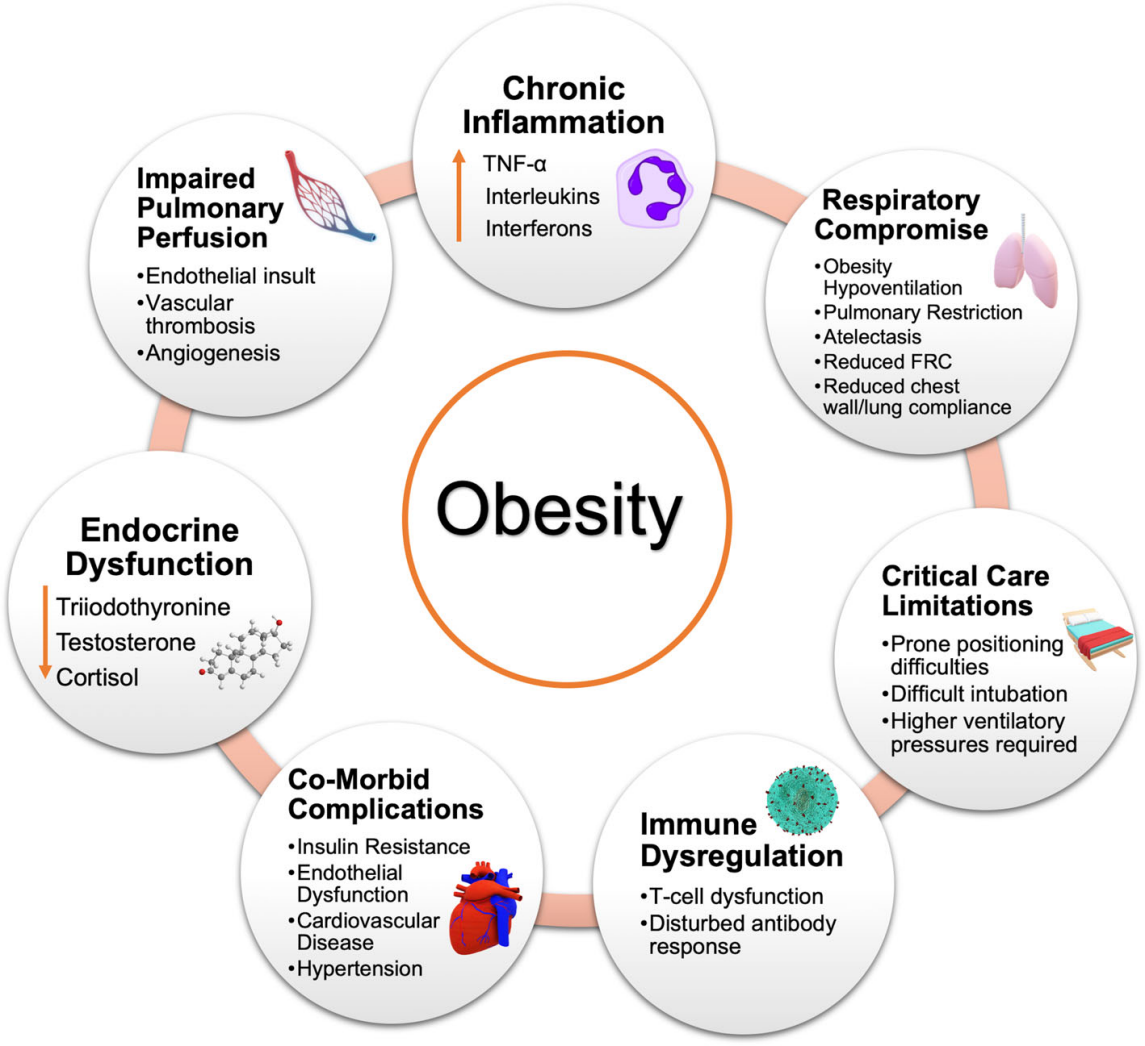
Potential mechanisms that augment risk of critical illness and death from COVID‐19 in people with obesity. There are a number of potential mechanisms by which obesity may influence adverse outcomes from COVID‐19. These include chronic inflammation, impairment of respiratory function and pulmonary perfusion, practical considerations when managing obese patients in critical care settings, immune dysregulation, metabolic and vascular complications of obesity and relative reductions in key hormones. TNF‐α, tumour necrosis factor alpha; FRC, functional residual capacity

Obesity affects respiratory function through a number of mechanisms (Figure 2), such as pulmonary restriction, ventilation‐perfusion mismatch and respiratory muscle fatigue, which can lead to reductions in ventilatory capacity and increases in the load placed upon it; furthermore, respiratory drive can be reduced. These complications increase the risk of obesity hypoventilation syndrome,^57^ particularly in those with severe obesity. Obesity is also frequently associated with obstructive sleep apnoea syndrome.^57^ It has been demonstrated that a 10% increase in weight is associated with a 32% increase in the apnoea‐hypopnoea index and a 6‐fold increase in the risk of moderate to severe obstructive sleep apnoea.^58^ Central obesity and excess visceral fat adversely affect both chest wall and lung compliance due to accumulation of fat deposits within the thorax and in the abdominal cavity.^59^ Movements of the diaphragm and chest wall are restricted by the thoracic wall and intra‐abdominal fat mass resulting in reduced resting volume of the lung (functional residual capacity).^59^ These changes in body mechanics and pulmonary function, coupled with a potentially dysfunctional immune system and increased likelihood of comorbidities in obesity, predispose patients with obesity to respiratory infections.

A previous study showed that intubation of individuals with obesity was more challenging than lean counterparts.^60^ Additionally, the propensity towards atelectasis in patients with obesity makes managing critical respiratory support challenging in these patients.^61^ It is accepted that in critical care prone‐positioning improves air entry to posterior lung regions and drainage of airway secretions, improving gas exchange and survival in patients with ARDS.^62^ Prone‐positioning has been recommended in mechanically ventilated COVID‐19 patients.^63^ Patients with obesity benefit from this technique although the mandatory regular turning of a sedated patient with obesity can be physically demanding for ICU staff. In a single centre ICU study it was shown that prone‐positioning in ARDS patients improved partial arterial oxygen pressure significantly more in patients with obesity than healthy‐weight patients.^64^ Additionally upper chest and pelvic supports are usually required in patients with obesity to avoid abdominal compression and the bed is frequently placed in the reverse Trendelenburg position to lessen transdiaphragmatic pressure and atelectasis.^50^

### 4.1 Obesity and pulmonary perfusion

Obesity and chronic inflammation are also closely related to endothelial dysfunction, which is both a contributor and consequence of pro‐inflammatory pathway activation. Host infection of SARS‐CoV‐2 occurs through the ACE2 receptor, which is expressed in multiple organs including the lungs, heart, kidneys, as well as the endothelial cells.^65^ SARS‐CoV‐2 has been shown to be capable of directly infecting engineered human blood vessel tissue cultures,^66^ and this observation was subsequently extended to a patient series where diffuse endothelial inflammation, dysfunction and apoptosis were seen on post‐mortem histology. In an autopsy study of the pulmonary pathobiology of COVID‐19 compared to H1N1, there were three distinctive angiocentric features of COVID‐19.^67^ The first was severe endothelial injury associated with intracellular SARS‐CoV‐2 virus and disrupted endothelial cell membranes; the second was widespread vascular thrombosis with microangiopathy and occlusion of alveolar capillaries; the third was significant new vessel growth through a mechanism of intussusceptive angiogenesis in the lungs from patients with COVID‐19. This provides a link between COVID‐19 and the complications of organ ischaemia, inflammation and pro‐thrombotic state driven by systemic microcirculatory impairment, which is likely to be compounded by the pre‐existing endothelial dysfunction associated with obesity and its related comorbidities (Figure 2).

## 5 OBESITY AND THROMBOTIC RISK

Obesity is a known risk factor for thrombotic disorders such as venous thromboembolism (VTE) including deep vein thrombosis and pulmonary embolism, cardiovascular disease and stroke, and an independent predictor of myocardial infarction irrespective of sex, age, and ethnicity. Obesity‐driven chronic inflammation and impaired fibrinolysis appear to be major effector mechanisms of thrombosis in obesity.^68^ It is thought that the pro‐inflammatory and hypofibrinolytic effects of obesity may be exacerbated by dysregulated expression and secretion of adipokines and microRNAs.

An early study from Wuhan reported that mortality from COVID‐19 was associated with significantly higher levels of D‐dimer and fibrin degradation product (FDP) and prolongation of prothrombin and activated partial thromboplastin times on admission compared to survivors^69^; 71.4% of non‐survivors compared to 0.6% survivors had disseminated intravascular coagulation. The reported incidence of VTE in COVID‐19 ranges up to 8% in general wards, up to 35% in the ICU setting, and up to 58% in consecutive autopsies in patients in whom VTE was not suspected before death (reviewed by Marietta et al).^70^ Intriguingly, it has been speculated that in patients with severe/critical COVID‐19 disease there develops a ‘consumptive fibrinolysis’ due to overwhelming levels of fibrin and misfolded proteins/necrotic tissue in the lung, suggesting a clinical paradox where plasmin formation can be either deleterious or beneficial in COVID‐19, but not at the same time.^71^ In hospitalized patients with COVID‐19, low molecular weight heparin (preferred) or unfractionated heparin at prophylactic doses for prevention of VTE is recommended by the World Health Organization^72^ and several scientific societies (reviewed by Marietta et al).^70^ There is also limited evidence of benefit from enhanced platelet inhibition treatment with aspirin, clopidogrel, tirofiban and the factor Xa inhibitor fondaparinux.^73^

## 6 OBESITY AND IMMUNE FUNCTION

Obesity is associated with increased production of inflammatory cytokines such as TNF‐α, interleukins and interferons that characterize chronic low‐grade inflammation, which impair immune responses, both innate and adaptive. A hyperinflammatory response in which there are raised levels of interleukins and TNF‐α has been associated with increased mortality from COVID‐19.^74^ The chronic inflammation in patients with obesity is speculated to be contributory to the observed increased mortality due to a potential enhancement of the inflammatory response to COVID‐19 infection and induced disturbances in T‐cell mediated immunity.^75^, ^76^ Indeed, obesity has been associated with increased activation of pro‐inflammatory T‐helper (Th‐) 1 and Th‐17 cells with reductions in anti‐inflammatory Th‐2 and regulatory T‐cells.^77^, ^78^ An elegant study by Misumi et al demonstrated that not only were memory T cell quantities increased in obesity, their function was disrupted leading to tissue destruction following viral infections.^79^ A more recent study of peripheral blood mononuclear cells showed enhancement in TNF‐ and Fas‐induced T cell‐apoptosis in patients with COVID‐19.^80^ The T‐cell response is increasingly being postulated as being pivotal in reducing susceptibility to and adversity from SARS‐CoV‐2^81^, ^82^, ^83^ and impaired T‐cell immunity may be key to obesity‐related detriment in relation to COVID‐19.

Obesity is characterized by adipose tissue remodelling,^84^ and pro‐inflammatory alteration of the adipokine profile.^85^ The resultant imbalance between pro‐ and anti‐inflammatory adipokines have been implicated as key to obesity being a major risk factor for acute lung injury.^85^, ^86^ Leptin, a pro‐inflammatory adipokine produced primarily by white adipocytes, is closely related to the immune system, playing a regulatory role in T‐cell activation and cytokine production.^87^ The impairment in host defence against pulmonary infections is thought to relate to leptin resistance resulting from prolonged hyperleptinaemia.^88^ Indeed, leptin resistance in T cells, natural killer (NK) cells and peripheral blood monocytes have been demonstrated in obesity.^89^, ^90^, ^91^, ^92^

Adiponectin has anti‐inflammatory properties and exerts favourable effects on insulin sensitivity.^65^ Reduced adiponectin levels have been observed among both patients of normal weight and obesity in the presence of cardiometabolic dysregulation.^93^ Adiponectin functions as a regulator of macrophage polarization from pro‐inflammatory M1 to anti‐inflammatory M2 macrophages,^94^ and low levels in obesity have been associated with adverse outcomes in patients with emphysema, asthma, and respiratory failure in the critical care setting.^95^ Adiponectin deficiency has also been demonstrated to increase lung inflammation and reduce clearance of apoptotic cells in animal models.^96^ It is of note that adiponectin levels in metabolically healthy individuals with obesity (MHO) are higher than those with impaired metabolic health^93^; it is the latter group that is predisposed to increased risk of pneumonia and worse outcome in COVID‐19.^97^

## 7 OBESITY AND THE ENDOCRINE SYSTEM IN COVID‐19

Adipose tissue is an active organ that plays an important role in several important physiological functions that are mediated through hormones and adipocytokines.^98^ Obesity is associated with several endocrine alterations that arise as a result of changes in the hypothalamic‐pituitary hormonal axes. These include hypogonadism, hypothyroidism and cortisol deficiency (Figure 2), which may have a role in mediating the adverse relationship between obesity and COVID‐19 outcomes.

### 7.1 Obesity and testosterone

Testosterone concentrations are lower in older men,^99^ and in men with severe obesity of all ages.^100^ While low testosterone is associated with reduced respiratory muscle activity and exercise capacity^101^ and higher levels of pro‐inflammatory cytokines,^102^ testosterone replacement therapy improves peak oxygen consumption^103^ and reduces cytokine levels.^102^ Therefore, the hypothesis arises that low testosterone in ageing men with obesity may have a role in the cytokine storm of SARS‐CoV‐2 infection leading to more severe disease. It is not yet known, however, if testosterone is beneficial in the treatment of COVID‐19 in men with obesity and low testosterone levels. Furthermore, a contrary hypothesis of testosterone‐driven COVID‐19 progression also exists.^104^ This is based on androgen receptor activation of a protease that is crucial for COVID‐19 viral spread.^105^ It is thus postulated that it is the higher testosterone levels observed in men that leads to their higher mortality outcomes. More research is needed to elucidate the exact relationship between testosterone and COVID‐19 before any definite conclusions can be drawn.

### 7.2 Obesity and thyroid function

The hypothalamic‐pituitary‐thyroid hormone axis may be affected by both obesity and significant illness. Conversely, the treatment of thyroid dysfunction may also be associated with weight gain.^106^ Obesity is associated with a rise in serum thyrotropin (thyroid stimulating hormone; TSH) level although thyroid hormones tend to remain stable or may even be mildly elevated.^107^ In illness thyroid function tends to be abnormal, typically leading to reduction in TSH and triiodothyronine (T3) levels in the initial phase. This dampening down of the hypothalamic‐pituitary‐thyroid pathway has historically been considered as an adaptive response to reduce metabolism and conserve energy. However, some experts have questioned this view as those with the lowest serum T3 levels have the worst outcomes.^108^ The reduction in T3 levels seems to mirror the rise in inflammatory cytokines seen in acute illness.^109^ Controversy continues as to whether thyroid hormone therapy has beneficial or detrimental effects in euthyroid individuals with obesity undergoing caloric deprivation and in euthyroid adult patients during non‐thyroidal illnesses.^110^ One randomized controlled trial of T3 therapy in patients with heart failure and low circulating T3 levels demonstrated a significant improvement in left ventricular ejection fraction and improvement in inflammatory markers.^111^ There is no data, as yet, to suggest that abnormal thyroid function, particularly in people with obesity infected with SARS‐CoV‐2, is associated with adverse outcomes. Therefore, it remains to be seen if patients with obesity and COVID‐19 infection and low serum T3 levels benefit from T3 supplementation.

### 7.3 Obesity and the glucocorticoid axis

The hypothalamic‐pituitary‐adrenal axis, which is intimately involved in the regulation of energy metabolism and bodyweight,^112^ may play an important role in the global rise in obesity. Hair cortisol levels, a novel non‐invasive parameter reflecting mean cortisol levels over several months, are increased in people with obesity compared to normal‐weight individuals.^113^, ^114^ The hypothalamic‐pituitary‐adrenal pathway may be affected by COVID‐19 infection. Autopsy studies performed on patients who died from the SARS virus in 2003 showed degeneration and necrosis of the adrenal cortical cells. In fact the coronavirus causing SARS was first identified in the adrenal glands, hinting towards a direct cytopathic effect of the virus. Hence it is likely that cortisol dynamics may be altered in patients with SARS (and possibly in patients with COVID‐19 too). One of the primary immune‐invasive strategies employed by the SARS‐CoV is to knock down the host's cortisol stress response.^115^ Antibodies produced by the host to counteract the virus, in turn, would unknowingly destroy the host ACTH (adrenocorticotrophic hormone), thereby blunting the cortisol rise. This would imply that all patients with SARS might have had underlying relative cortisol insufficiency.^116^ However, observational data on circulating cortisol levels in patients with COVID‐19 are scarce. A recent cohort study reported higher levels of cortisol in hospitalized COVID‐19 patients on admission compared to those without COVID‐19.^117^ Elevated cortisol level was associated with reduced survival and was a marker of disease severity. The results of ongoing trials of corticosteroid therapy in patients hospitalized with COVID‐19 infection will be insightful. Indeed, an early report from the RECOVERY trial has shown that oral or intravenous dexamethasone 6 mg daily reduced deaths by one‐third in ventilated patients (rate ratio 0.64) and by one fifth in other patients receiving oxygen only (RR 0.82).^118^ However, dexamethasone treatment in this context is likely to work via its anti‐inflammatory and immunomodulatory actions rather than any possible effects on the pathophysiology of COVID‐19.

## 8 OBESITY AND MICRONUTRIENTS

The role of trace elements and vitamins in COVID‐19 has drawn the attention of the scientific community and the lay public. A large body of data show that vitamins, including vitamins A, B6, B12, C, D, E, and folate, trace elements, including zinc, iron, selenium, magnesium, and copper, and omega‐3 fatty acids play important and complementary roles in supporting the immune system.^119^ Vitamin D deficiency, in particular, has been mooted as a potential contributor to susceptibility to COVID‐19.^120^ Prevalence of vitamin D deficiency is 35% greater in people with obesity compared to healthy‐weight individuals and is associated with obesity irrespective of age, latitude, cut‐offs to define vitamin D deficiency and the Human Development Index of the study location.^121^ Rates of vitamin D deficiency are markedly high in people with severe obesity and potential bariatric surgical candidates in northern latitudes.^122^ Further research into screening and treating vitamin D deficiency to reduce COVID‐19 risk is warranted. While some small observational studies, which did not control for potential confounding factors such as BMI or underlying health issues, have reported that inadequately‐treated vitamin D deficiency is associated with increased risk of COVID‐19, a large‐scale population study of UK Biobank participants reported that vitamin D was weakly associated with COVID‐19 infection on univariable analysis, but not after adjustment for confounders.^123^ A rapid review from Oxford found no clinical evidence related to vitamin D deficiency predisposing to COVID‐19, nor evidence in favour of supplementation for preventing or treating COVID‐19.^124^ An evidence review from the National Institute for Health and Care Excellence concluded that there is no evidence to support taking vitamin D supplements to specifically prevent or treat COVID‐19.^125^ However, all people should continue to follow public health advice for the general population on vitamin D supplementation,^126^ to prevent vitamin D deficiency and maintain bone and muscle health during the COVID‐19 pandemic, irrespective of any possible link with respiratory infection.

## 9 OBESITY, DRUGS AND VACCINES

There remains a need to develop effective treatment and a vaccine for SARS‐CoV‐2. Obesity poses potential difficulties for both. It is often overlooked as a cause of suboptimal treatment in infectious diseases owing to its adverse influence on pharmacokinetic and pharmacodynamic properties of drugs, as well as their efficacy and safety.^127^ Even as obesity becomes a global phenomenon, the effects obesity has on drug absorption, distribution, metabolism and clearance are not completely understood,^128^ and dosing recommendations for patients with obesity are often not provided in pharmaceutical drug information sheets. Lower vaccine efficacy has been reported in people with obesity following the H1N1 pandemic.^38^ While there was no significant difference in antibody titres between people with obesity and healthy‐weight individuals 1 month after H1N1 vaccination, people with obesity had a 4‐fold or greater decline in antibody titres compared to healthy‐weight individuals at 12 months post‐vaccination.^129^ Obesity is associated with T cell dysfunction and the impaired response means a proportion of individuals with obesity remains at risk of influenza despite vaccination.^130^ A similar risk with a future COVID‐19 vaccine would be concerning. Importantly, the typical participant in the recently published early phase trials of vaccines against SARS‐CoV‐2 was normal weight.^131^, ^132^, ^133^ Although these trials have shown promising results, applying their findings to populations with high background prevalence of obesity – 40% in the United States, 29% in England and 13% globally – carries a degree of uncertainty which may hopefully be addressed in Phase 3 trials.

## 10 BENEFITS OF WEIGHT LOSS

The benefits of weight‐loss on complications of obesity, insulin resistance and systemic inflammation are well‐documented, especially after bariatric surgery.^134^, ^135^, ^136^ However, even short‐term small amounts of weight‐loss can show substantial metabolic benefits.^137^ This is particularly important during the COVID‐19 pandemic; it is essential that weight‐loss is encouraged as a public health intervention, albeit even if only small changes are feasible. Healthcare professionals should discuss weight‐loss goals and methods with patients with obesity; this can be done using virtual and telephonic consultations with the patient's own weighing scale as a reference. Patients should be encouraged to make lifestyle changes focused on nutritional modification, calorie restriction and augmentation of exercise; individualized approaches are likely to yield most benefit. Bariatric surgery should be considered where clinically appropriate as it leads to improvements in obesity‐related metabolic comorbidities such as diabetes, hypertension, dyslipidaemia, insulin resistance and inflammation.^136^, ^138^ It slows the atherosclerotic process and reduces cardiovascular and all‐cause mortality. Recent data have demonstrated regression of the microvascular complications of obesity and diabetes, including regeneration of small nerve fibres after bariatric surgery.^138^ A recent consensus statement provides guidance on bariatric surgery during the COVID‐19 pandemic.^139^ With the current public awareness about the risks of death from COVID‐19 in persons with obesity, there may be an added motivation for patients to partake in weight‐loss programs. Conversely, this public awareness can provide a source of anxiety for these patients and therefore the benefits of any amount of weight‐loss should be emphasized to patients to aid their general well‐being.

## 11 FUTURE RESEARCH DIRECTIONS

Our current understanding of obesity as an independent risk factor for severity of COVID‐19 is borne out of observational studies. Further research is necessary to better elucidate the cellular and molecular mechanisms that underlie this increased risk. Urgent research is also required into pharmacotherapeutics for COVID‐19 in people with obesity to better understand efficacy and failure of antiviral drugs, immunotherapies and vaccines. Whether significant weight loss in people with obesity, particularly massive weight loss after bariatric surgery, influences outcomes of COVID‐19 remains to be seen and needs further study. Ultimately, research into the biological, psychological, socio‐cultural and economic drivers of obesity and its management are essential for building societal resilience to future pandemics.

## 12 CONCLUSION

Obesity is a risk factor in viral pandemics and infected patients with obesity have a worse disease prognosis. COVID‐19 is no exception and a report has just been published by Public Health England on the associations of excess weight with COVID‐19.^140^ During pandemics individuals with obesity should be included as one of the clinically vulnerable groups, especially those with morbid obesity (BMI > 40 kg/m^2^). Clinical trials of medicinal products should emphasize the inclusion of people with obesity to better understand the effects of obesity on pharmacokinetics. Further research into vaccination regimes is necessary to achieve and maintain better immune response in patients with obesity to subsequent virus exposure.

## CONFLICT OF INTEREST

Dr Le Roux reports grants from Science Foundation Ireland, grants from Health Research Board, during the conduct of the study; other from NovoNordisk, other from GI Dynamics, personal fees from Eli Lilly, grants and personal fees from Johnson and Johnson, personal fees from Sanofi Aventis, personal fees from Astra Zeneca, personal fees from Janssen, personal fees from Bristol‐Myers Squibb, personal fees from Boehringer‐Ingelheim, outside the submitted work. All other authors have nothing to disclose.
